# Lane and Heat Draw Have Little Effect on Placings and Progression in Olympic and IAAF World Championship 800 m Running

**DOI:** 10.3389/fspor.2019.00019

**Published:** 2019-09-03

**Authors:** Brian Hanley, Arturo Casado, Andrew Renfree

**Affiliations:** ^1^Carnegie School of Sport, Leeds Beckett University, Leeds, United Kingdom; ^2^Faculty of Health Sciences, Isabel I University, Burgos, Spain; ^3^Institute of Sport and Exercise Science, University of Worcester, Worcester, United Kingdom

**Keywords:** coaching, elite-standard athletes, endurance, race tactics, track and field

## Abstract

The aim of this study was to establish whether the lane and heat draw influenced placings and progression in world-class 800-m track running. Finishing positions and times of 1,086 performances at the Olympic Games and IAAF World Championships between 1999 and 2017 were obtained. Mean finishing and season's best times (SB), as well as placings and progression rates, were found for each heat number and for the inner (Lanes 1 and 2), middle (Lanes 3–6), and outer lanes (Lanes 7 and 8). In the qualifying heats and semi-finals, the theoretically expected number of fastest losers (non-automatic qualifiers) per heat was compared with the actual number. One-way ANOVA with Bonferroni *post-hoc* tests were conducted to compare finishing times between lane and heat numbers across rounds. With regard to the order of heats, there were no differences between finishing times in either the qualifying heats or semi-final rounds for men; in the women's event, only Semi-final 3 was the quickest, but still did not have higher progression rates. SB times did not differ between heats within each round, highlighting the fair distribution of athletes. Progression rates for each lane during the qualifying heats ranged between 36 and 52% (men) and between 49 and 61% (women), close to the expected ranges of 45 and 55%, respectively. The middle lanes were quicker in the seeded semi-finals and finals only. Men in the outer lanes fared slightly worse and should focus on achieving the optimal tactical position after breaking from lanes. The IAAF could reconsider how they allocate seeded lanes in the later rounds by switching the fifth and sixth fastest athletes from the outer to the inner lanes. Regarding the heat draw, athletes mostly did not take advantage of knowing previous performances from earlier races, and probably focused on achieving an automatic qualifying position instead. However, the fastest losers in the women's last semi-final were faster and showed that benefitting from the heat draw is possible with tactical coaching.

## Introduction

The 800 m is the shortest middle-distance event held at the Olympic Games and International Association of Athletics Federations (IAAF) World Championships, and differs from other distance races in that the first bend is run in lanes (IAAF, 2017a). Recent research has shown that very fast starting paces are adopted in global championship 800 m races (Hanley et al., 2019), and the effects of lane allocation for the first bend could be a factor affecting this. The lane allocated to each athlete in the semi-finals and final is based on performances achieved over the course of the year (including performances in earlier rounds of those championships) so that the four athletes with the fastest times are randomly drawn in the middle four lanes, with the next two fastest randomly drawn in Lanes 7 and 8, and the two slowest randomly drawn in Lanes 1 and 2 (IAAF, 2017a); the four slowest were randomly drawn across Lanes 1, 2, 7, and 8 until 2009 (IAAF, 1997, 2008).

Unlike the semi-finals and final, in the first round (the “qualifying heats”), the lane draw is by lot, and could therefore confer an advantage on those drawn in the middle four lanes as previous research suggests running speed is more limited in the inner lanes because of constraints on the forces generated by the inside leg (Taboga et al., 2016). Indeed, the IAAF discontinued indoor 200 m races in 2005 (that are held on six-lane 200-m tracks) because those allocated to the outer lanes had too great an advantage (Taboga et al., 2016). Additionally, the very outer lanes on an outdoor track (lanes 7 and 8) are considered disadvantageous because athletes starting in those lanes cannot easily see other athletes to pace themselves in the very early stages (Morgan, 2016). Although the proportion of the race run in lanes is relatively short, the need to break from lanes on the back straight means that deciding on the best route to the 200-m distance, where the next bend occurs, is highly important because athletes want to minimize total distance run, avoid being blocked in, and gain possible drafting benefits (Casado and Renfree, 2018). In 800 m championship racing, athletes can improve their chances of a middle lane draw by running a season's best time in the previous round(s), but no study to date has examined what effect lane draw has on placings and progression in 800 m running in terms of qualifying for later rounds or winning medals in world-class competition. New research comparing placings and progression and finishing times for the inner, middle, and outer lanes will therefore highlight whether such an advantage does exist, and therefore provide information that could be used by the IAAF to consider the fairness of the current allocation rules.

In many sports that adopt head-to-head competition structures (e.g., swimming and rowing; FINA, 2017; FISA, 2017), athletes are seeded before the competition begins to try to ensure that the best athletes reach the final. Such an approach is also taken in 800 m running, where the heat draw (i.e., which heat each athlete runs in) is based on performances achieved during the qualification period, with athletes allocated in a zigzag distribution (Table 1). This distribution of athletes is intended to achieve parity across heats, although exceptions are sometimes made when drawing heats to separate athletes from the same nation (IAAF, 2017a). Seeding in the semi-finals and final is based on these same performances, except for those athletes who run faster during the earlier rounds (as for the lane draw). Progress from the qualifying heats to the semi-finals, and from the semi-finals to the final, can be achieved either through a high-enough finishing position (usually the top two) or by having one of the best non-automatic qualifying finishing times; athletes qualifying by time rather than position are often referred to as “fastest losers” (IAAF, 2017b). Very occasionally, athletes who fail to qualify can progress if the Jury of Appeal decides they have been impeded unfairly (IAAF, 2017a). Knowing the finishing times of other athletes in earlier heats could give those in the last heat a competitive advantage (IAAF, 2017a) as they could theoretically pace themselves to achieve the required time and qualify as fastest losers (provided not too many rivals in the same race run faster times). However, as with the lane draw, whether the last heat does in fact produce the greatest proportion of fastest losers has not been examined, and therefore it is not established whether heat draw has any effect on placings and progression in qualifying (i.e., for the fastest loser positions). It has also not been ascertained whether seeding for lanes (in the first qualifying round) or heats produces equally weighted races in global championships, and therefore whether the process of allocating athletes by qualifying time works in achieving fair competition. Knowing whether there are differences between lanes or heats could inform coaches and athletes of suitable tactics to adopt to take advantage of the draw, or minimize any potential drawbacks. Similarly, if the draw is potentially unfair, the IAAF could reconsider the processes adopted for allocating heats and lanes. The aim of this study was to establish whether the draws for heats and lanes have an effect on success in 800 m racing. It was hypothesized that athletes running in the middle four lanes would achieve better placings and progression in the seeded semi-finals and finals, but that there would be no difference for placings or progression in the unseeded qualifying heats, or between the randomly allocated heat numbers in the qualifying heats and semi-finals.

**Table 1:** Example of how 48 athletes would be drawn into six qualifying heats.

**Heat allocation**	**Athlete ranking by SB**							
A	1	12	13	24	25	36	37	48
B	2	11	14	23	26	35	38	47
C	3	10	15	22	27	34	39	46
D	4	9	16	21	28	33	40	45
E	5	8	17	20	29	32	41	44
F	6	7	18	19	30	31	42	43

*Athletes are placed in qualifying heats (and semi-finals) using the order of seeding by season's best times (SB) in a zigzag distribution (IAAF, 2017a), so that the mean ranking per heat is equal. The allocation can deviate from this model if athletes representing the same nation are drawn in a heat together. The actual order in which the heats are run is drawn by lots*.

## Materials and Methods

### Research Approval

The protocol was approved by the Carnegie School of Sport Research Ethics Committee with the requirement for informed consent waived as the study analyzed publicly available data only. The study was conducted in accordance with the recognized ethical standards of the Declaration of Helsinki.

### Participants

Official electronic finishing times and positions of all competitors in the men's and women's 800 m competitions at the Olympic Games and IAAF World Championships between 1999 and 2017 were obtained from the open-access IAAF website (IAAF, 2018) as shown in Supplementary Data. In each of the championships analyzed, a round of qualifying heats was held, with the number of qualifying heats varying depending on the number of entrants. Three semi-finals were normally held for each 800 m event (men and women), but because only two semi-finals were held in a small number of championships (i.e., 1999, 2000, and 2013 for women; 2001 for both men and women), these championships were omitted. A total of 1086 championship performances (303 men: 664 performances; 206 women: 422 performances), with many athletes competing in several championships, were analyzed. The performances of 61 men and 52 women in the qualifying heats, and 14 men and six women in the semi-finals, were removed as outliers as their finishing times were more than 1.5 times the interquartile range (IQR) from the median of the scores (Hanley, 2016). The results of 13 men and three women who were disqualified, and eight men and four women who did not finish during the qualifying heats, were excluded from the analysis. In the semi-finals, the results of five men and five women who did not finish, and four men who were disqualified, were not included in the analysis of that round. The results of three men and one woman who qualified for the semi-finals via the Jury of Appeal were included for analysis in the semi-finals and final (as appropriate), but not in the qualifying heats. Similarly, the results of two men who qualified for the final (in 2009) by appeal were included for analysis in the qualifying heats and final. The season's best time (SB) for each analyzed athlete was obtained from the IAAF website (IAAF, 2018), and their finishing times for each round calculated as a percentage of SB (“SB%”). Sixteen men and five women who were analyzed had no SB recorded before the championships.

### Data Analysis

The study was designed as observational research in describing placings and progression per ordered lane and heat. In most championships, the stadium had an eight-lane track; on those occasions when the track had nine lanes, the inside lane was typically vacated and thus for those occasions Lane 2 was considered Lane 1, etc. On the very rare occasions that nine athletes competed in a race and doubling-up in a single lane occurred, both athletes' performances were counted for that lane. For the analysis of effect of lane draw, the number of qualifiers from each lane (comprising automatic qualifiers and fastest losers, but not those who progressed by appeal) in the qualifying heats and semi-finals were measured, as were the number of medalists per lane in the final.

The number of qualifying heats per championship varied from six to nine (men) and five to eight (women). Accordingly, the number of athletes qualifying as fastest losers varied so that, when added to the automatic qualifiers, 24 progressed to the semi-finals. Exceptions occurred in the men's event in 2000, 2001, and 2012 when appeals meant that 25 took part in the semi-finals, and 2017, when one athlete dropped out of the competition before the semi-finals. The single exception in the women's events was in 2009 when 25 took part in the semi-finals because one athlete progressed by appeal. To account for the variance in the number of qualifying heats, the theoretically “expected” number of fastest losers per heat was calculated; for example, if there were eight fastest losers qualifying from eight qualifying heats, the expected number per heat was one. The expected total across all championships for each qualifying heat was then found (i.e., Heat 1, Heat 2, etc.) and compared with the actual number of qualifiers from those heats. All semi-finals had a format of three separate races, with the top two finishers advancing as automatic qualifiers and two fastest losers qualifying across all three races. Because there was a set number of automatic qualifiers per qualifying heat and semi-final, the performances of the fastest losers were analyzed rather than the automatic qualifiers, but the mean times of all athletes in each heat were also measured to indicate overall race quality, and to allow comparisons between heats regarding whether even distribution of athlete ability occurred.

### Statistics

Results are reported as mean ± one standard deviation (SD). One-way analysis of variance (ANOVA) with Bonferroni *post-hoc* tests were conducted to compare mean finishing and season's best times between qualifying heat numbers and semi-final numbers for both fastest losers and all athletes. One-way ANOVA with Bonferroni *post-hoc* tests were also used to compare finishing times, SBs and SB% between the “inner” lanes (Lanes 1 and 2), “middle” lanes (Lanes 3, 4, 5, and 6), and “outer” lanes (Lanes 7 and 8). Effect sizes for differences found were calculated using Cohen's *d* (Cohen, 1988), rounded to two decimal places and considered to be either trivial (*d* < 0.20), small (0.21–0.60), moderate (0.61–1.20), large (1.21–2.00), or very large (> 2.01) (Hopkins et al., 2009). Pearson's chi-squared test of association (χ^2^) compared observed counts of categorical data (e.g., qualified or did not qualify, won a medal, or did not) between the inner, middle, and outer lanes. Similarly, to analyze progression rates from qualifying heats, the number of fastest losers from the first half of the qualifying heats were grouped, and compared with the number from the second half using Pearson's chi-squared test of association. Progression rates in the qualifying heats were compared using the first and second halves of the draw because of the disparity in the number of qualifying heats between championships (e.g., Heat 5 in 2004 was the middle heat of nine, whereas in 2005 it was the second last heat). In those instances where an odd number of qualifying heats were held (as occurred in four men's and three women's championships), there was one qualifying heat more included in the first half than in the second. An alpha level of 5% was used for all tests; 95% confidence intervals (95% CI) were also calculated (for the chi-squared test, this was the 95% CI of the unadjusted odds ratio; Field, 2009).

## Results

**Figure 1:**
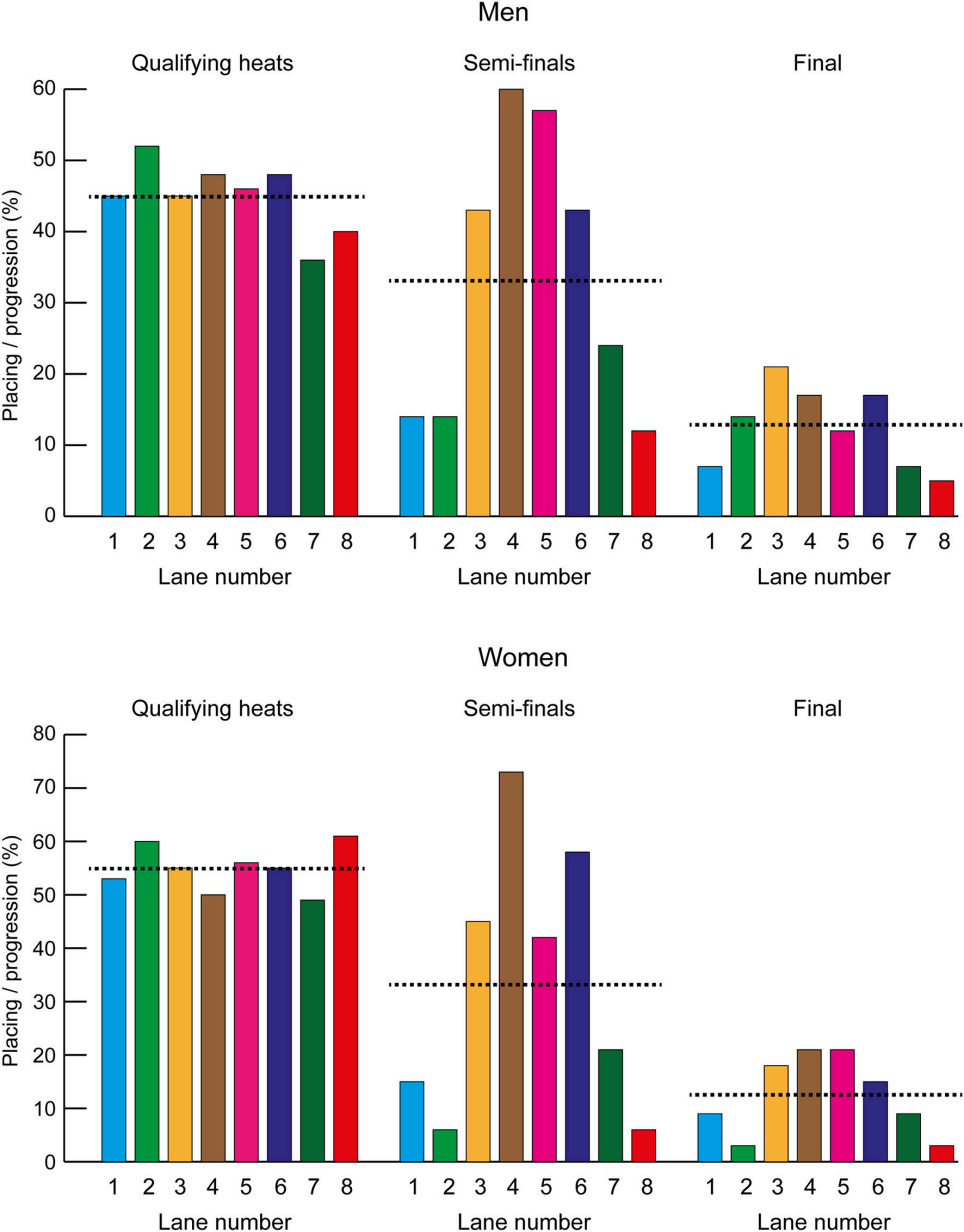
Placings and progression rates (%) for each lane based on the proportion of athletes running in that lane who qualified for the next round, or who won medals in the final. The expected value (shown as a dotted line) refers to the percentage of athletes who would be expected to qualify from each lane or win a medal if randomly allocated.

Figure 1 shows the placings and progression for each lane based on the proportion of athletes running in that lane who qualified for the next round, or who won medals in the final, compared with the expected rate per lane (which equaled the mean of the actual rates found across all lanes). During the qualifying heats, there were no differences in the women's event between the progression rates of those running in the middle lanes compared with either the inner or outer lanes, but in the men's qualifying heats the progression rate was higher in the middle lanes and inner lanes than in the outer lanes [middle vs. outer: $\chi_{\left(1\right)}^{2}$ = 4.00, *p* = 0.045, 95% CI: 1.01–2.04; inner vs. outer: $\chi_{\left(1\right)}^{2}$ = 4.09, *p* = 0.043, 95% CI: 1.01–2.35]. In the men's semi-finals, those in the middle lanes were more likely to qualify [middle vs. inner lanes: $\chi_{\left(1\right)}^{2}$ = 31.77, *p* < 0.001, 95% CI: 3.15–12.31; middle vs. outer lanes: $\chi_{\left(1\right)}^{2}$ = 24.52, *p* < 0.001, 95% CI: 2.46–8.77], as was the case in the women's semi-finals [middle vs. inner lanes: $\chi_{\left(1\right)}^{2}$ = 35.43, *p* < 0.001, 95% CI: 4.30–23.79; middle vs. outer lanes: $\chi_{\left(1\right)}^{2}$ = 31.12, *p* < 0.001, 95% CI: 3.54–16.89]. In the men's finals, those in the middle lanes were more likely to win a medal than those in the outer lanes [men: $\chi_{\left(1\right)}^{2}$ = 5.65, *p* = 0.017, 95% CI: 1.17–8.51], but not more so than those in the inner lanes. In the women's finals, those in the middle lanes were more likely to win a medal than those in either the inner or outer lanes [both: $\chi_{\left(1\right)}^{2}$ = 5.84, *p* = 0.016, 95% CI: 1.20–10.89].

Table 2 shows the mean finishing and SB times for those running in the inner, middle, and outer lanes, with annotations of any differences found. In all tables and the text below, differences between running times have been annotated only when the effect size was moderate or larger and the 95% CI did not cross zero. There were no differences found between inner, middle and outer lanes for SB% in any round for women and, in the men's event, only between those in the middle lanes (101.5 ± 1.2%) and inner lanes (100.8 ± 1.1%) in the semi-finals (*p* < 0.001, *d* = 0.62, 95% CI: 0.28–1.05).

**Table 2:** The mean finishing and SB times (min:s) (±SD) for the inner (Lanes 1 and 2), middle (Lanes 3–6) and outer lanes (Lanes 7 and 8) in each round.

	**Men**			**Women**		
	**Inner**	**Middle**	**Outer**	**Inner**	**Middle**	**Outer**
Finishing time (min:s)						
Qualifying heats	1:47.32 (±1.29)	1:47.39 (±1.43)	1:47.48 (±1.23)	2:02.17 (±1.93)	2:02.47 (±2.47)	2:02.28 (±2.18)
Semi-finals	1:46.58^a^ (±1.01)	1:45.66^a^^b^ (±1.16)	1:46.55^b^ (±1.23)	2:00.87^a^ (±1.36)	1:59.54^a^^b^ (±1.35)	2:00.65^b^ (±1.53)
Final	1:45.33 (±1.24)	1:44.92^b^ (±1.44)	1:45.89^b^ (±1.69)	1:59.04^a^ (±1.21)	1:57.68^a^^b^ (±1.77)	1:59.05^b^ (±1.75)
Season's best time (min:s)						
Qualifying heats	1:45.47 (±1.22)	1:45.57 (±1.65)	1:45.66 (±1.65)	2:00.17 (±1.61)	2:00.14 (±2.29)	2:00.03 (±1.64)
Semi-finals	1:45.69^a^^c^ (±0.63)	1:44.09^a^^b^ (±0.66)	1:45.27^b^^c^ (±0.82)	2:00.37^a^^c^ (±0.74)	1:58.31^a^^b^ (±1.17)	1:59.86^b^^c^ (±0.63)
Final	1:44.71^a^ (±0.62)	1:43.64^a^^b^ (±0.67)	1:44.38^b^ (±0.45)	1:58.65^a^ (±0.54)	1:57.08^a^^b^ (±1.00)	1:58.46^b^ (±0.82)

a *Significant difference between middle and inner lanes*.

b *Significant difference between middle and outer lanes*.

c *Significant difference between inner and outer lanes*.

*Significant differences (p < 0.05) between running times have been annotated only when the effect size was moderate or larger (d ≥ 0.61) and the 95% CI did not cross zero*.

Tables 3, 4 show the progression of the fastest losers by qualifying heat and semi-finals, as well as the mean times run by the fastest losers and all athletes in each ordered heat. In both men's and women's qualifying heats, there was no difference in qualifying progression rates between the first half and second half of races. In the men's event, there were no differences between mean finishing times in either the qualifying heats or semi-finals for either fastest losers or all athletes, although men competing in Semi-final 1 were more likely to qualify as fastest losers than those in Semi-final 3 [$\chi_{\left(1\right)}^{2}$ = 5.87, *p* = 0.015, 95% CI: 1.21–10.45]. In the women's event, there were no differences in the qualifying heats, but Semi-final 3 was quicker than both Semi-final 1 (*p* = 0.028, *d* = 1.79, 95% CI: 0.09–1.92) and Semi-final 2 (*p* = 0.017, *d* = 1.40, 95% CI: 0.15–1.74); however, no differences were found regarding likelihood of qualifying as fastest losers.

**Table 3:** Progression of fastest losers by qualifying heat and semi-final in the men's event; the number of qualifiers (as fastest losers) is shown alongside the expected number from that heat number (“exp.”).

**Heat**	**Occurrences**	**Qualifiers (exp.)**	**Fastest losers (min:s)**	**All athletes (min:s)**
Heats				
1	14	7 (12)	1:46.62 (±0.69)	1:47.44 (±1.33)
2	14	13 (12)	1:46.40 (±0.47)	1:47.22 (±1.17)
3	14	17 (12)	1:46.28 (±0.35)	1:47.18 (±1.66)
4	14	13 (12)	1:46.12 (±0.53)	1:47.25 (±1.22)
5	14	14 (12)	1:46.53 (±0.45)	1:47.51 (±1.39)
6	14	6 (12)	1:46.45 (±0.61)	1:47.78 (±1.38)
7	8	7 (6)	1:46.67 (±0.34)	1:47.42 (±1.20)
8	5	6 (5)	1:46.07 (±0.26)	1:47.35 (±1.33)
9	1	0 (1)	–	1:47.57 (±0.78)
Semi-finals				
1	14	14 (9)	1:44.92 (±0.50)	1:46.00 (±1.21)
2	14	9 (9)	1:45.12 (±0.35)	1:46.09 (±1.13)
3	14	5 (9)	1:45.38 (±0.35)	1:46.23 (±1.32)

*The mean times (±SD) for the fastest losers, as well as all athletes in each heat, are also shown. All expected values were rounded to the nearest integer*.

**Table 4:** Progression of fastest losers by qualifying heat and semi-final in the women's races; the number of qualifiers (as fastest losers) is shown alongside the expected number from that heat number (“exp.”).

**Heat**	**Occurrences**	**Qualifiers (exp.)**	**Fastest losers (min:s)**	**All athletes (min:s)**
Heats				
1	11	9 (12)	2:01.18 (±0.82)	2:02.22 (±1.87)
2	11	14 (12)	2:01.24 (±1.10)	2:02.20 (±2.21)
3	11	7 (12)	2:01.43 (±1.51)	2:03.07 (±2.74)
4	11	11 (12)	2:01.10 (±1.69)	2:02.15 (±2.08)
5	11	13 (12)	2:01.07 (±0.69)	2:02.72 (±2.57)
6	8	13 (9)	2:01.24 (±1.16)	2:01.78 (±2.13)
7	1	0 (1)	–	2:02.49 (±0.90)
8	1	0 (1)	–	2:01.23 (±1.89)
Semi-finals				
1	11	5 (7)	1:59.27 (±0.37)	2:00.13 (±1.30)
2	11	8 (7)	1:59.21 (±0.71)	1:59.91 (±1.30)
3	11	9 (7)	1:58.26 (±0.64)	2:00.41 (±1.89)

*The mean times (±SD) for the fastest losers, as well as all athletes in each heat, are also shown. All expected values were rounded to the nearest integer*.

The mean SBs for the fastest losers and all athletes per heat are shown in Table 5. There were no differences found between SBs in either the qualifying heats or semi-finals for either men or women.

**Table 5:** The mean SB times (min:s) (±SD) for the fastest losers, as well as all athletes, in each heat.

**Heat**	**Fastest losers (min:s)**	**All athletes (min:s)**	**Fastest losers (min:s)**	**All athletes (min:s)**
	**Men**		**Women**	
Qualifying heats				
1	1:46.56 (±3.07)	1:45.68 (±1.90)	1:59.86 (±1.08)	2:00.20 (±1.99)
2	1:45.27 (±0.95)	1:45.51 (±1.53)	1:59.43 (±1.07)	1:59.95 (±1.99)
3	1:45.88 (±1.30)	1:45.55 (±1.63)	1:59.84 (±0.87)	2:00.12 (±2.20)
4	1:45.26 (±1.22)	1:45.51 (±1.43)	2:00.43 (±1.28)	2:00.03 (±1.84)
5	1:45.32 (±1.39)	1:45.53 (±1.50)	2:00.28 (±1.23)	2:00.07 (±1.52)
6	1:45.67 (±1.38)	1:45.58 (±1.48)	2:00.45 (±0.79)	2:00.27 (±2.41)
7	1:45.65 (±1.20)	1:45.67 (±1.60)	–	2:01.33 (±2.97)
8	1:45.94 (±1.33)	1:45.60 (±1.33)	–	1:59.93 (±1.88)
9	–	1:45.57 (±1.35)	–	–
Semi-finals				
1	1:44.68 (±0.94)	1:44.81 (±0.94)	1:59.08 (±0.85)	1:59.23 (±1.23)
2	1:44.38 (±0.95)	1:44.73 (±1.06)	1:58.48 (±1.14)	1:59.18 (±1.44)
3	1:44.46 (±0.68)	1:44.81 (±0.99)	1:58.43 (±1.30)	1:59.23 (±1.32)

## Discussion

The aim of this study was to establish whether the draws for heats and lanes have an effect on placings and progression in 800 m championship racing. The fact that lane draw (in the qualifying heats) and heat draw (qualifying heats and semi-finals) is by lot, with no differences in SBs found, allows for a robust analysis of the effects of those draws. Regarding the lane draw, there was no difference in qualification rates or finishing times between the inner, middle and outer lanes during the randomly drawn first-round qualifying heats for women, although men in the outer lanes had lower progression rates than those in the inner and middle lanes (by ~10%). This was despite no difference in finishing times, SBs or SB% between lane groupings, and suggests that some men were unable to overcome the disadvantages of running in the outer lanes, and so the hypothesis that there would be no difference between lanes for placings or progression in the unseeded qualifying heats was rejected for the men's event. The lack of a difference in finishing times could reflect how achieving qualification can be a matter of very close finishes (Hanley et al., 2019), and tiny details of pacing can matter. As hypothesized, there were higher placings and progression rates for the middle four lanes during the semi-finals and final, which was unsurprising as the effects are biased because the highest-ranked athletes were drawn in those lanes, and their SBs were indeed faster than those in the inner and outer lanes. Apart from one exception, there were no differences in SB% between lane groupings, showing that athletes ran times relative to their ability regardless of their allocated lane.

Starting in the outer lanes prevents athletes from seeing their rivals who could be used as external references for pacing (Renfree et al., 2014a), but also allows them to choose a better position when breaking as there are few if any opponents on their outside. Conversely, running in the inner positions allows athletes to see their opponents but might be blocked by them as they converge inwards after the breaking point, although the inner lanes were slower than the outer lanes during the semi-finals only (which was not unexpected as the outer lanes have been allocated to faster athletes since 2009). Each lane thus has its own advantages and disadvantages, and though the random allocation of lanes that occurs in the qualifying heats is fair, being able to see other competitors in the inner lanes might outweigh the disadvantage of the tighter bend for men. Furthermore, no differences were found in the probability of achieving a medal during the men's finals between athletes in the inner and middle lanes, even though the middle lane athletes had run faster SBs. The concern that running in the inner lanes might hinder 800 m athletes is therefore unjustified as athletes run the other three bends in the inner lanes to achieve the shortest total distance in any case, and are therefore accustomed to their curvature. Additionally, the slower pace adopted compared with 200 and 400 m races might reduce any impact of running in the inner lanes. It is possible that those athletes who doubled up in a lane competed with each other for the inside position within their lane, necessitating a faster start than normal, but these incidences were very rare. Instead, the very inside lane was often vacated, either because fewer than eight athletes competed in any particular race (although never in the semi-finals) or because a nine-lane track was used.

Because the first 100 m, which is run in lanes, represents one eighth of the total race distance, tactical positioning is a very important aspect of championship racing (Casado and Renfree, 2018), and athletes should consider potential tactical options. Whereas world-class athletes drawn in the outer lanes during the heats do not need to worry unduly about their starting lane, as, like in the later rounds, it is usually those with the fastest season's best times who qualify (Renfree et al., 2014b), those of lesser ability need to reduce any potential disadvantage of starting in the outer lanes. These athletes should try to experience multiple races before a major championship, as practicing running in the outer lanes can be useful when learning to take the shortest realistic path when breaking to the inside (Martin and Coe, 1997). Championship racing is, however, quite different from Diamond League competition because of the absence of pacemakers (Filipas et al., 2018) and athletes should develop tactical judgment when breaking to avoid being boxed in. Athletes might break for the inside earlier on the back straight when a headwind is blowing because of possible drafting benefits (Casado and Renfree, 2018), but those athletes in the very outer lanes should consider the extra distance run (Martin and Coe, 1997). Indeed, those men in the outer lanes (who were less likely to progress from the qualifying heats) might have made poor tactical decisions when breaking to the inside, resulting in more total distance run. Ultimately, athletes should focus on achieving the optimal tactical position at 200 and 400 m as this has a greater effect on qualifying probability (Casado and Renfree, 2018). Based on these novel results, the IAAF could reconsider the current performance-based allocation of lanes in 800 m outdoor championship events, with athletes ranked fifth and sixth randomly allocated to the inner lanes, rather than the outer lanes at present. In cases where athletes drop out of the competition after lanes have been drawn (e.g., before the semi-finals), athletes could be moved to fill empty lanes to move them closer together, especially to avoid isolating athletes in the outer lanes.

The draw for the qualifying heats and semi-finals is designed to achieve equally weighted races so that the highest ranked athletes avoid each other and qualify for the next round. This study found that the seeding of qualifying heats and semi-finals in this manner did indeed achieve a fair distribution of competitors' abilities as no differences were found for either all athletes or the fastest losers within a race; this part of our hypothesis was therefore accepted. Indeed, the mean times for fastest losers in the qualifying heats were within such a narrow range (1:46.07–1:46.67 for men and 2:01.07–2:01.43 for women) that they provide coaches with very strong indicators of typical 800 m performances needed to progress. Although, from a tactical viewpoint, it is considered advantageous to run in later heats (IAAF, 2017a), this study found that athletes did not take advantage of knowing what times previous fastest losers had run (the progression rate for men's Heat 6 was approximately half that of earlier qualifying heats), notwithstanding that those in qualifying Heat 1 had relatively poor progression rates, possibly because they had no previous heat times to base their pacing on. By contrast, it was noticeable in the men's event that half of all fastest losers in the semi-finals qualified from the first race, and on the one occasion that women had eight qualifying heats, no fastest losers qualified from the last two heats. There are a number of reasons why most athletes in the later races did not benefit from knowing the current fastest loser standings: first, they might not have known other athletes' times as the duration between races is relatively short, and athletes have to focus on their own race; second, it might be too difficult to pace oneself to such a specific time with few immediate sources of feedback; and third, middle-distance athletes have been found to be more concerned with finishing position, rather than time, even during the qualifying heats (Hanley and Hettinga, 2018). This makes sense given that, in the semi-finals, at least one third-placed athlete will not qualify, no matter how good their finishing time is, and reiterates the importance of achieving an optimal tactical position after breaking from lanes. This was supported by the finding that the fastest losers in the women's Semi-final 3 were faster than those in the prior semi-finals, but the numbers qualifying were not greater. It is also possible that athletes in the later heats calculate that they are unlikely to beat earlier fastest loser times, and focus on trying to achieve an automatic qualifying time. As with the lane draw, there were no clear benefits to being drawn in any particular heat (even if there potentially could be), and athletes should similarly not worry about this aspect of the championship structure at the expense of focusing on the race itself. The data used for this study were taken from championship results and, although this provides high ecological validity, are therefore limited to the numbers of participants who took part. Because the analysis undertaken involved dividing these athletes by heat and lane, the numbers available for statistical analysis are relatively low. As low sample sizes can lead to underpowered studies and a consequent increase in the possibility of Type II errors (Cohen, 1992), it is possible that differences occurred that were not detected. Future studies should consider adding to the data analyzed in this study with those found in future IAAF World Championships and Olympic Games.

## Conclusions

This study analyzed men's and women's 800 m races at global athletics championships and found that there was little effect of lane or heat draw on eventual placings and progression, especially for women. In the randomly drawn qualifying heats, men in the outer lanes fared slightly worse, possibly because of greater difficulties in early pacing, and coaches should work with their athletes to practice pace management in the first 200 m in particular. Athletes should therefore adopt the most appropriate tactics when breaking from their lane (a balance between running the shortest distance, avoiding being boxed in, and obtaining possible drafting and pacing benefits from the pack). There was no clear evidence of athletes in later heats taking advantage of knowing other athletes' finishing times (e.g., more men qualified from the first semi-final than the last one, rather than the other way around), but this does not mean that this is not possible, and coaches could try to pass on useful information about approximate target times, or whether focusing on an automatic qualifying position is the priority. Seeding for the qualifying heats and semi-finals works in terms of distributing athletes evenly and fairly, but the IAAF could consider allocating the inner lanes to faster athletes rather than the outer lanes as the inner lanes appeared to present less of a disadvantage. It should be noted though that the analysis might be underpowered with an increased possibility of Type II errors, and hence future studies should consider adding to these data already recorded at global championships.

## Data Availability

All datasets generated for this study are included in the manuscript/Supplementary Files.

## Author Contributions

BH, AC, and AR conceptualized and designed the study, wrote the manuscript, and read and approved the final manuscript. BH conducted the data collection and analyses and created tables. All authors read and approved the final manuscript.

### Conflict of Interest Statement

The authors declare that the research was conducted in the absence of any commercial or financial relationships that could be construed as a potential conflict of interest.
